# Unraveling complexity in changing mental health care towards person-centered care

**DOI:** 10.3389/fpsyt.2023.1250856

**Published:** 2023-09-14

**Authors:** Karin Lorenz-Artz, Joyce Bierbooms, Inge Bongers

**Affiliations:** ^1^Tranzo, Tilburg School of Social and Behavioral Sciences, Tilburg University, Tilburg, Netherlands; ^2^Mental Health Care Institute, Eindhoven, Netherlands

**Keywords:** person-centered care, open dialogue, transformation, mental health care, implementation

## Abstract

**Background:**

Mental health care (MHC) needs to shift towards person-centered care to better meet people’s individual needs. Open Dialogue (OD) is well-aligned with this perspective and brings it into practice. This study focuses on exploring the change process within a pilot project involving three MHC teams as they transition to a person-centered OD practice. Our aim is to identify and reflect on the challenges faced by MHC professionals in adopting person-centered care, and shedding light on the underlying complexity of these challenges. By gaining a better understanding of these obstacles, we hope to contribute to the adoption of the person-centered approach in MHC practice.

**Methods:**

Our research employed a qualitative design, involving a total of 14 semi-structured interviews with MHC professionals who were either trained in OD, OD trainees, or MHC professionals without OD training. To analyze the data, we utilized a hybrid approach that combined deductive – and inductive thematic analysis.

**Results:**

We identified four distinctive challenges: (1) understanding and knowledge transfer, (2) (inter)personal process, (3) emotional discomfort, and (4) the need for multi-stakeholder participation and support. In practice, these challenges intersect and the appearance of and relationships between these challenges are not linear or disentangleable.

**Conclusion:**

Upon careful consideration of these interdependent challenges, it became evident that embedding a person-centered approach like OD brings about systemic change, leading to an unfamiliar situation X. The research findings indicated that understanding and conveying the concept of person-centered care in practical settings poses significant challenges. The field of knowledge management helps to capture the complexity of understanding and transferring this knowledge. The change process necessitates an (inter)personal process and elicits emotional discomfort, as person-centered OD practice confronts a deeply entrenched paradigm in MHC. Achieving a shared understanding of person-centered care requires dedicated time and attention, while introducing this approach prompts broader discussions on underlying values and human rights in MHC. Current implementation efforts may underestimate or overlook these underlying values, but initiating an open dialogue can serve as an initial step in addressing the complexities.

## Introduction

Mental health care (MHC) needs to shift towards a more person-centered approach, which encompasses a holistic and relational view on mental illness (1–8). Several authors suggest that this approach enhances the quality of care, better meets person’s needs (9, 10) and emphasizes the vital role that the social network can play as participants in the care process (11). In addition to these expected benefits, the shift towards person-centered care has been fueled by severe criticism on psychiatry for its narrow and pathologizing perspective on individuals who are experiencing psychological distress (7, 8, 12–14). Its positivistic and reductionist tendencies are being condemned for dehumanizing and stigmatizing those struggling with mental health, labeling them as abnormal (8, 15). Furthermore, the value-justice aspect of MHC is being challenged, emphasizing the importance of treating clients with dignity, respect and compassion (10, 14, 16). MHC clients frequently feel unheard and do not receive the help they need (2, 17, 18). This criticism on psychiatry pertains to the ethical considerations as well as the treatment outcome (10) and calls for a radical change in MHC (19, 20).

However, the concept of person-centered care is complex and involves the multiple levels within MHC (e.g., health system, organization, and provider/individual level) (10). It necessitates a radical shift in MHC, involving the modification of its norms and expectations, and ultimately its culture (19). If current MHC truly wants to deliver person-centered care and wants to prevent it from being just “a buzz phrase” and remaining an aspiration, then recognizing and addressing the level of complexity that exists within this change process that obstructs the needed radical change is essential (9, 21, 22).

This means that the radical change does not only involve practical applications but relates to deeply entrenched underlying values and ethical considerations as well. The central theme that connects the existing definitions of person-centered care is the ethical principle of recognizing clients in treatment as individuals deserving to be treated with personhood. In simple terms, people living with severe mental illness are people and citizens. This immediately emphasizes the significance of freedom and human rights (7, 17, 23). Many MHC professionals claim to already provide individualized and respectful care, considering clients’ values and preferences. However, real-world practice often falls short of these claims (16, 24). Beyond treatment outcomes (10), the justification for person-centered care is rooted in its perception as an inherent human right, irrespective of achieved results. Consequently, assessing the significance of a treatment intervention should not only involve reviewing existing evidence but, more importantly, evaluating its potential to uphold and reinforce human rights (7, 14), grounded in community inclusion, self-determination and giving a voice to those affected (14, 24, 25).

Zooming into the implications in practice, this call for radically changing MHC implies that psychiatry needs to abandon these positivistic and reductionist tendencies and undergo a radical shift towards person-centered practice with focus on helping individuals to live a meaningful life, in contrast to setting treatment goals that are largely dictated by professionals (8, 26, 27). Recovery is to be seen as a personal and subjective experience, rather than a treatment outcome and as a personal “journey” which can be supported, but not controlled by MHC professionals (2). This new focus requires a change in interaction style and power relation between professional and client (8, 19), meaning that MHC practice should be built on equal partnership, hope-promoting and facilitating self-determination (27). The voice of the client becomes a participatory agent (7) in which it is about personhood (each individual is a unique person with their own potential) instead of patient-role (associated with vulnerability and dependence) (19).

Open Dialogue (OD) appears to align well with this new focus and perspective (1, 4, 5, 11) and confronts the established deeply entrenched paradigm in MHC. The OD approach involves a paradigm shift that challenges established norms and power structures (28, 29). OD is a profound multi-layered approach – including a philosophy, therapeutic attitude and skills, and organizational structure – which gives concrete form to the multi-layered concept of person-centered care and enables the involvement of different treatment approaches, professional backgrounds and MHC services to adapt to client needs (14, 30–34). Within this approach mental problems are seen as a resonance in the interpersonal and should be considered as a shared interactional problem instead of an individual problem. Subsequently, OD shifts focus from a solution-oriented perspective to a relation-oriented perspective. OD goes beyond learning new skills (the “doing”) as it also involves a personal change in the vision, values, and attitude of the professionals (the “being”) (35, 36).

It is considered a value-based practice, due to its foundation in the belief that essential values should be transparently articulated and defined, as they shape our perspective, actions, and capacity to establish affirmative and supportive interpersonal bonds (37). OD explicitly outlines its fundamental values, which encompass unconditional warmth, authenticity, and openness (32). These values are not congruent with mainstream MHC, which prioritizes the client-MHC professional relationship, where the MHC professional diagnoses and administers treatments. This system follows a professional bureaucracy structure, with a focus on experts’ decisions and authority, resource and risk management, standards adherence, and enabling experts’ skills and knowledge (38). In contrast, OD suggests that professionals reflect on what strikes a chord and comes forth from within them. They permit themselves to be influenced more profoundly, sharing personal experiences from a genuine vulnerability, which might differ from what professionals trained in distance and proximity are accustomed to Lorenz-Artz et al. (34). These OD values resonate with the fundamental qualities of person-centered approaches upheld by MHC professionals. These include the capacity for understanding, empathy, authenticity, acceptance without conditions, and the promotion of empowerment, allowing those who receive the services to exercise autonomy in their lives (39). Additionally, these values align with the four main ethical principles (beneficence, nonmaleficence, autonomy, and justice) (40, 41) and concretely define the essential attributes that MHC professionals should embody.

As a manifestation of person-centered care, the OD approach also confronts the established deeply entrenched paradigm in mental health care. The OD approach involves a paradigm shift that challenges established norms and power structures (28).OD embodies aforementioned values, exemplified by the adage “nothing about me, without me” OD refers to both shared-sense-making and shared-decision-making (34). Literature on person-centered care regards shared decision-making as a critical aspect, and various skills are delineated to aid professionals in effectively communicating in a person-centered manner: e.g., open interview skills training, learning how to pick up on cues relating to emotional distress, empowering language, exploring family and social issues with sensitivity and enquiring about the meaning of particular symptoms to persons (22, 24). This aligns with OD, which offers specific psychotherapeutic skills to professionals that draw from both dialogic work and systems therapy (30, 42, 43). In the literature, the OD approach is often explained by its seven guiding principles: (1) immediate help, (2) social network perspective, (3) flexibility and mobility, (4) responsibility, (5) psychological continuity, (6) tolerance of uncertainty, and (7) dialogism [e.g., (30)]. These seven principles give guidance to put the concept into practice and cover the themes that structurally come forward in the literature defining person-centered care: sharing power; sharing responsibility; therapeutic alliance; clients as a person; shared decision making; biopsychosocial; provider as a person; coordinated care; access; continuity of care (7, 10, 19).

In this study, we aimed to unravel the paradigm shift towards person-centered care based on a thorough analysis of a pilot project of three MHC teams in a transition process towards adopting OD. We strived to pinpoint the distinctive challenges that arose during this real-life change process. The central research question was what challenges MHC professionals encountered when adopting the person-centered OD care philosophy. The emphasis was not on the pragmatic aspects of implementing the new approach, such as the organizational requirements. In the discussion section, we reflect on these challenges by unraveling the complexity underlying these interrelated challenges in order to elucidate the nature of these challenges. In doing so, we hope to contribute to the adoption of the person-centered approach, such as OD, in practice.

## Method

### Setting

This study took place within a 2-year pilot program, introducing the OD approach in three Flexible Assertive Community Treatment (FACT) teams within GGz Eindhoven and the Kempen (GGzE), a Dutch mental health care institution based in the Southern part of the Netherlands. These teams applied the FACT model, which is a team-based approach with its focus on assertive outreach, consisting of a multi-disciplinary team of mental health professionals, including psychiatrists, psychologists, social workers, occupational therapists, and peer-support workers. The FACT teams offer treatment to clients with severe mental illness (SMI) for whom traditional mental health services may be less suitable (44). Each of the three pilot teams provides care to over 200 clients with approximately 20–30 new clients being referred every year. For clients who are relatively stable they provide individual case management and home visits from Monday to Friday from 8 a.m. to 6 p.m. For clients with more severe needs, they have a shared caseload with a full treatment approach, allowing care to be increased and decreased relatively quickly as needed. In a crisis situation that requires admission, the crisis team is called in.

The introduction of OD within these FACT teams was aimed at enhancing care in a person-centered approach. Explaining the comprehensive OD approach and its practical implications for the FACT teams in this study exceeds the intended scope of this study. However, to provide a stronger sense of the setting, we highlighted certain OD aspects that give substance to person-centered care in Appendix I.

At the end of 2017, seven FACT professionals from various FACT teams successfully completed the postgraduate training program called “Peer-supported Open Dialogue, Social Network and Relationship Skills” at the Academy of Peer-supported Open Dialogue (APOD) in the UK. For the purpose of this study, we will refer to these professionals as “OD professionals”. Subsequently, these OD professionals formed an OD pilot team, transitioning from the FACT method to fully embracing the OD approach.

In 2019, an additional four FACT professionals from the two FACT teams participating in this pilot underwent training and concurrently introduced OD within their respective FACT teams. We will refer to this group of professionals undergoing training as “OD trainees”. Within these two FACT teams, the OD trainees organized OD network treatment sessions upon request, in addition to delivering regular FACT care. The remaining colleagues who continued to provide FACT care are referred to as “FACT professionals” in this study.

Because it is not within the scope of this study to provide a detailed description of each action in the implementation process, we described in Appendix II specific actions related to the introduction of OD that appeared to have influenced the adoption of OD based on the perspectives of professionals who participated in the study. So, to be transparent, we elaborate in Appendix II upon the applied implementation strategies in order to provide background for a clearer understanding of the study’s findings.

### Design

For this qualitative study, an instrumental case design has been applied. The case study approach allows to study program-based reforms of services in detail in a real-life context (45). It is an instrumental case (46), because the purpose of the study is to gain a broader insight into the phenomenon of the needed transition of the mental health care, based on the pilot in which OD is introduced.

The data collection consists of semi-structured interviews. We used a hybrid approach, both deductive – and inductive thematic analyses were used (47). This strategy capitalizes on the advantages of both techniques to offer a more holistic understanding of the data. It enriches the research findings by incorporating established theoretical understanding while also revealing novel aspects that could enhance the current knowledge (48). Given the existing knowledge about change processes and OD, but the limited understanding of the challenges faced by mental health care professionals in this particular transition (29), it is important to apply both analysis approaches. Both the data collection and data analysis were carried out by two researchers. One researcher is OD trained and has previously worked in a clinical setting providing crisis care for people with severe mental illness. The other researcher is a behavioral scientist and did not do the OD training. Both researchers have experience conducting (qualitative) implementation research in the MHC setting. The study was approved by the Dutch Ethical Review Board of Tilburg School of Social and Behavioral Sciences, Tilburg University (REF EC-2019.EX113).

### Sampling & data collection procedure

#### Participants and recruitment

To ensure that the different perspectives of OD professionals, OD trainees and FACT professionals in the case are included, participants were recruited from all three pilot teams through purposive sampling with maximum variation in experience with OD practice, professional background, attitudes towards and experience with the OD approach (49). All professionals of the three pilot teams, including professionals with a positive, negative, or neutral attitude towards the OD approach were eligible. On request of the researcher, the department manager, who was the project leader of the OD pilot and completed the OD training, made a list of professionals to participate in this study. Four OD professionals from the OD team and four FACT professionals from the two FACT teams involved in the pilot, were recruited. In addition, all four OD trainees and the department’s two managers (one of whom is OD trained) were approached using the critical case sampling strategy. They have been chosen based on their specific position in the process and are considered critical for understanding the phenomena – transition of mental health care – studied (49).

All 14 recruited eligible professionals received an information letter from the researcher with the invitation to participate and request to respond within 14-days. All agreed on participating and signed an informed consent form, after which the interviews were planned.

During the interviews, it became apparent that the questions posed regarding developmental issues led to numerous personal statements, which is also evident from the result section. Nevertheless, the impact on the participants themselves was assumed to be inconsiderable, as their personal experiences were shared within the pilot teams independently of the present study. Additionally, participation in the study was entirely voluntary, and participants could end their involvement at any time without providing negative consequences.

#### Semi-structured interviews

Within the 2-years pilot program, a total of, 14 semi-structured interviews of 1–1.5-h were conducted in August and September 2019 on-site at the pilot teams. All interviews were conducted by two researchers. A topic list was used as a memory aid for the researchers during the interviews, to ensure all relevant topics to answer the research question were covered. The topic list contained different themes related to the research question and was based on literature about the Normalization Process Theory (NPT), conditions related to social innovations and basic concepts of OD [e.g., (50–54)]: such as coherence, cognitive participation, involvement of colleagues, received support and experienced struggles in the process. This literature assisted in being preemptively aware of the process steps involved in such a change process and the potential challenges that professionals may encounter. Four topic lists were used: one for the group OD professionals, one for the OD trainees, one for the FACT professionals and one for the managers. The same themes were addressed in the topic lists, but the questions were adapted to the backgrounds of the participants in order to approach the themes from different perspectives.

### Data analyses

All interviews were audio-recorded with the interviewees’ permission. All audio recordings were transcribed verbatim and analyzed using a renowned qualitative data analysis program called Atlas.ti (www.atlasti.com, accessed on 9-3-2023). We used a reflexive and hybrid approach, by analyzing through Braun and Clarke’s six phases of thematic analysis (55) in a both deductive and inductive manner (47). The six phases are: familiarization with the data, generating initial codes, searching for themes, reviewing theses, defining themes, and producing the final report. In the following paragraphs we describe these six phases linearly, although in the analyses we moved back and forth between phases as the analysis developed (55). We used theory-led analyses, without forcing data into a rigid theoretical framework.

After familiarization, by transcribing the audio data, reading and re-reading the data and note taking, we used a hybrid approach with a deductive an inductive technique to generate initial coding. We identified in this stage semantic themes, summarized the content of the data, and captured the surface meaning which reflects what was explicitly said (56). Deductive codes were based on literature about constraining conditions of social innovation implementation [e.g., (52–54)] and OD elements (50). When a relevant fragment did not fit within one of these codes, a new code was added (inductive). Two interviews were coded independently by two researchers, after which the results were compared with each other. Text fragments encoded differently were discussed. There were only minor differences. For instance, one researcher chose shorter text fragments, while the other researcher preferred longer text fragments to preserve more context within the text fragment. Another example is that one researcher also coded text fragments that were beyond the scope of the central research question, whereas the other researcher refrained from doing so. The decision was made to select slightly longer text fragments, but only those that fell within the scope of the central question. Subsequently, the interviews were split between the two researchers, and they only conferred with each other about fragments of text when there was uncertainty. At the end of this phase, preliminary results were presented to members of the pilot to ensure the different perspectives were accurately portrayed and the researcher’s interpretations were trustworthy (57). The members had no further feedback and recognized themselves in the results. This indicated that we were on the right track with the analysis, which we considered a green light to proceed to the next phase of analysis.

As a next step in the initial coding, we employed the NPT as an analytical framework and used the four stages (coherence, cognitive participation, collective action and reflexive monitoring) and its underlying mechanisms (58) as initial codes, to uncover and untangle and thereby gain better understanding of the taken actions and the complexities that have emerged during the transformative process to person-centered care within the pilot. The appearance and relations between the found aspects were not linear but helped to focus our attention on how the transformative process evolved in pilot program (59). We have used the text fragments that were selected in the previous analysis round. These have been re-coded deductively from the NPT perspective, using thematic analysis (47, 55). In this phase, we have used a detailed matrix in which we reinterpreted and operationalized all four domains and constructs of the NPT (Appendix III).

After generating this set of initial codes with deductive and inductive technique, we developed inductively in a reflective and iterative process latent themes. To effectively apply this approach, we thoroughly studied methods and research examples to comprehend its practical implications. The NPT takes an interpretive stance, evaluating themes based on their prevalence and relevance – their capacity to capture crucial aspects related to the research question. However, the frequency of a theme in the dataset does not inherently determine its significance. Instead, a theme’s importance arises from its essential contribution to addressing the primary research question (55, 60–65). These latent themes go beyond what was explicitly said, revealing the underlying ideas, assumptions, and conceptualizations within the data (66). We listed potential themes and reviewed, refined and finally defined them. We defined four (latent) themes referred to as “challenges” in the results section of this study: (1) understanding and knowledge transfer, (2) (inter)personal process, (3) emotional discomfort, and (4) the need for multi-stakeholder participation and support. We have decided not to retrospectively map these inductively generated latent themes onto NPT constructs because it would be needed to force the themes into the framework and thus do insufficient justice to the practice in which the themes played throughout the process.

Note that in all the stages of analyses the three different pilot teams and professional backgrounds (OD professionals, OD trainees and FACT professionals) were taken into account by evaluating variations and similarities among the different teams and backgrounds. In addition, any doubts during the stages of analysis were discussed with a second researcher.

## Results

In this result section we elaborate on the complicating phenomena that have surfaced during this transformative change process towards the new OD practice and aim to pinpoint the distinctive challenges that arose during this change process. Note that in practice the transformative process was dynamic and contingent and challenges in the change process emerged simultaneously. We found the following distinctive challenges: (1) understanding and knowledge transfer, (2) (inter)personal process, (3) emotional discomfort, and (4) the need for multi-stakeholder participation and support.

### Understanding and knowledge transfer

One of the challenges that emerged in practice was the difficulty for OD professionals and OD trainees to understand OD, resulting in an underestimation of the profound and far-reaching impact of applying OD on themselves and the organization of care. It only became gradually clear what the meaning and far-reaching implication of the OD approach entails during the OD training and their first experiences with the OD approach in practice. This insufficient insight into what exactly OD entails prior to the start and during the pilot ensued a ripple effect, including too little preparation for the pilot. This led to a quick decision on the managerial level to change strategy during the year prior to the pilot: bringing OD professionals together in one OD team rather than spreading the OD approach across teams in the departments. This sudden change in strategy caught professionals off guard and had a negative impact on their support, creating a less receptive context where some expressed concerns that individuals undergoing OD training might face the same fate as the OD professionals who were removed from their own team. Another complicating consequence of this insufficient insight prior to the start was that the OD team had to pull out all the stops to function as a new team in a very short time, converting this approach and translating it into their own practice and further mastering this approach.

“I also found OD very interesting. Fortunately, I didn't do it myself. Because then colleagues were suddenly plucked out of all the teams and a separate team was formed. While beforehand, it wasn't clear at all… suddenly those people were really ripped out of the team. That was a total surprise for us, maybe for them too, but I don't know” (FACT professional).

The OD team considered the first pilot year being very complicated for three reasons. First, the introduction of OD implicated on practical level major changes in the working procedures, forming a new team of which half was still on training, taking over the careload of the prior team, and coping with a high turnover rate of staff within the OD team. Second, although OD had offered guidance on providing person-centered care, there was still a lack of clarity at the conceptual level, requiring the translation and implementation of its concepts into practical solutions for effective application in real-world practice. Frequent discussions were required to arrive at an appropriate policy and working method within the Dutch context, including determining the extent to which person-centeredness should be implemented in practice. OD professionals and OD trainees explained that OD needs to adjust to the context and vice versa, since OD is not in line with the current system. Third, related to the process level, an OD professional used the phrase “implementing is experimenting, “suggesting that the process should be approached with a “learning by doing” mentality, given the lack of a predefined blueprint for a “normal” course of action. To navigate this liminal space and integrate OD’s person-centered values, the management stimulated the OD professionals to push the limits, to make mistakes and to learn from them. This required searching for the golden mean between giving space and offering the demanded structure and continuous reflection on whether the struggles were inherent in the process or whether adjustment was needed. In order to gradually enhance and integrate the concepts of OD in practice, as well as to address any emerging misunderstandings in a timely manner, it was considered imperative to engage in ongoing dialogue among professionals throughout the change process. As such, this change process demanded creativity and flexibility from the professionals involved.

“We started like crazy, all super excited, and then the struggles come. We didn't really know whether those struggles are part of it or not. Is that unique to the team or is this normal. Are we doing it right or are we not doing it right?” (OD professional).

Simultaneously, OD trainees and FACT professionals from the two FACT pilot teams experienced a lack of communication from the side of the OD team. In addition to the struggle to understand the concepts of OD, they have experienced the OD team as closed, not transparent, and inward-looking during the whole pilot. For them, it remained unclear throughout the pilot period how OD is being implemented by the OD team and questions related to whether and how person-centered care is feasible for the client group with SMI remained unanswered. For example, what would happen when clients do not take the lead, which colleagues believe is often the case within the client group with SMI and concerns raised about clients being neglected. Another question was, how to continuously involve clients, with the underlying adage “nothing about me, without me” when it is already complicated to have them show up for an annual treatment plan meeting. Similar to the lack of contact between the OD team and the FACT teams, there was also little contact between the two FACT teams, which was also attributed to the high workload. Though, all professionals expressed a desire for increased communication among the pilot teams and to have more opportunities to connect with other OD professionals and OD trainees across the country.

“Well, what I particularly notice is that they are very inward-focused as a team. And I believe the strength of OD could be that, when it comes to implementation, you should collaborate much more with people from other teams, for instance. Make much more use of each other. And I feel that they are still somewhat, well, I'm not saying they're being self-absorbed, but they are oriented inward. More inward than outward” (OD trainee).

In terms of knowledge transfer, during the initial stages of the pilot and throughout its first year, the primary focus was on explicit knowledge transfer. OD professionals and OD trainees explained that they made efforts to explicitly explain how they pursue person-centered care. However, this approach often resulted in linguistic ambiguity, as the same words were used with different meanings. For instance, when it came to concepts like not setting goals for clients, actively listening to them, and following their process, (FACT) professionals often believed they were already incorporating these practices and saw no difference.

“Yes, I basically think that it won't be any different from the FACT team. And then I think about connecting with the care that's needed, and as little care as possible, that somebody can function as best as they can with what they have” (FACT professional).

“So all kinds of themes that belong to OD have already been discussed within the team. Also, about what is that dialogue, and how modest you actually are as a social worker. And how well do you actually listen. Well, things that you think we all do already, now everyone has started to think again. So that is a lot of fun” (OD trainee).

Furthermore, the idea of following the client’s process without steering in a particular direction sometimes led to the misconception that not having predetermined treatment goals or directly focusing on solutions amounted to doing nothing. In this situation, OD professionals and OD trainees tended to try and persuade others of the differences, but this approach proved to be counterproductive. Another related complication mentioned in the knowledge transfer process was the challenge of articulating certain aspects of OD. This was particularly true for implicit and tacit knowledge, which is better conveyed through experiential learning than verbal communication. Consequently, FACT professionals were encouraged to participate in network meetings to have the opportunity to experience the OD approach firsthand and understand the practical implications of the explicit knowledge being transferred.

“One of the biggest struggles I personally face is that capturing the essence of OD in words is incredibly difficult. When you try to put it into words, there is a risk, let me put it this way, that it becomes such a soft, hippie-like story, where we all go back to the 70s, anti-psychiatry. Some people get the chills” (OD professional).

“Truly understanding is challenging. And it really resides at an experiential level. So, with OD, I think you really have to experience, to understand what makes it different. And only then can you say … and even then, you still don't fully grasp it, but yeah” (OD professional).

### (Inter)personal process

Besides being difficult to understand and to transfer, this shift towards person-centered care is also considered a profound personal change process. OD professionals and OD trainees use phrases as “OD is a way of life” or “a way of love” to express that OD involves a transformation as a human being. OD professionals and OD trainees noted that they underestimated the impact of OD on themselves and added that this also applied to the OD training: the intensity, the effect of the used methods within the training and the role of mindfulness.

“I've also seen a number of people break down there because it gets so close what you don't expect. A number of people were away from home for the first time with many colleagues. You've already discussed things (in the training) that are very intimate, but just not enough, and then suddenly that exercise with family constellation is there” (OD trainee).

Within this personal change process, OD professionals and OD trainees gradually came to understand what OD implied. During the transition towards greater understanding, it appears that each group of the same training year underwent its own unique process. OD professionals and OD trainees noted that in the pilot program, professionals who were at similar stages of training tended to converge and were in contact, as they were experiencing similar developmental challenges that were different from those faced by professionals in other stages of training (please refer to Appendix II for additional training information). As an example, during the second pilot year, OD trainees were invited to attend expert meetings with the OD professionals, but they discovered that the topics discussed did not meet their needs.

“The interaction between the OD team and the people attending the training is limited. You can also notice that the groups going to England undergo their own development as a group” (OD professional).

In similar vein, each FACT team starting OD underwent its own process. For example there was a difference in the response of colleagues in the two FACT teams: one team being more supportive and enthusiastic about the introduction of OD and the other team was enthusiastic about OD but more conservative and skeptical about the manner in which OD was introduced and taught which overshadowed their enthusiasm. In the latter team, the coexistence of a skeptical perspective and the increasing enthusiasm of OD trainees resulted in friction, unlike in the other FACT team.

“The team was also very enthusiastic, which is quite nice. There are other teams. But our colleagues are very enthusiastic and contribute constructively” (OD trainee).

“Back then, they were already saying, ‘Well, just be careful, because you might come back all spaced out and brainwashed.’ And that felt very constricting” (OD trainee).

Interestingly, the OD professionals, OD trainees and several FACT professionals held divergent views on the necessity of undergoing such rigorous training and the desirability of the resulting transformative process. OD professionals and OD trainees promoted the importance of a profound training and personal transformation to internalize person-centered values. In contrast, certain FACT professionals expressed skepticism regarding the approach taken to teach OD and whether individuals should undergo personal changes. Certain FACT professionals expressed reservations regarding the use of therapeutic techniques, such as employing family constellations for the personal development of professionals themselves, without their explicit request for help. They highlighted that subjecting individuals to diverse therapeutic interventions, coupled with group pressure and the intensity of a four five-day residential training, without adequate prior information, can result in a risky combination. Some FACT professionals experienced the OD professionals and OD trainees as inwards and secretive, which made them feel unsafe and insecure.

“What I think is that it's a group that is very closed off. Very closed off. People visit each other. They call each other for advice. It feels like something you can't become part of. It feels like a tight-knit group. And sure, if you spend weeks together, 24/7, you also become closer. But it feels like we are OD and you are FACT. And maybe that's not the intention at all, but that's how it feels… it remains very exclusive and secretive. Somehow, there is a lack of open communication. And it feels like you're on the outside” (FACT professional).

In some cases, individuals used terms like “sectarian” and “brainwash” to caution against the worst-case scenario where participants may lose their ability to think critically. While OD professionals argued that such intensive training is necessary to achieve the necessary profound change and to fully understand the essence of OD. They added that this process requires continuous self-work and self-care and acknowledged that is it important to keep thinking critically. Holding frequent peer-to-peer consultations within teams as well as having a mixed team with multiple perspectives to keep an open attitude is considered necessary.

“Because what was definitely very clear is that the methodology entails the professional truly adopting a different approach within the process of providing assistance, different from how they have been trained up until that point. So, the training for it is essential” (OD professional).

“What happens is that you spend a week there, 24/7, and they use techniques from psychotherapy and brainwashing techniques. And when you're there 24/7, no matter how strong you are, you get caught up in it. There's a lot of group pressure, uh, group feeling. The way it is presented, I am absolutely against… yes, brainwashing is a big word, it's the part where you become very receptive to it, where there are brainwashing-like activities. While they give psychotherapy-like exercises, even though you have no question, no problem, and no description. So, all of this together leads to you having a different mindset” (FACT professional).

### Emotional discomfort

In addition to and partly due to the intricacy of comprehending and conveying OD, and the personal developmental processes involved, the introduction of OD into the organization also elicited emotional discomfort. This sense of discomfort was considered common in the liminal space between the old and new situation but could also be attributed to the resistance against the manner in which OD was introduced, its confrontation with deeply ingrained paradigms, and the multitude of changes inherent in the transitional process.

Commencing with the finding that the OD professionals and some OD trainees tended to introduce OD with a palpable and unwavering conviction, driven by their belief that care needs to be fundamentally different. The proposed changes were perceived by some FACT professionals as an attack, suggesting that their current work practices were inadequate or incorrect. For example, the adage “Nothing about me, without me” represents a powerful assertion that professionals should not talk about clients behind their backs. Furthermore, the emphasis on personal transformation in the context of OD has led some FACT professionals to dismiss it as a “tree-hugging ideology.” This perception may also be influenced by the role of mindfulness, which is not commonly integrated into Dutch MHC practices except as a therapeutic approach for clients. The combination of strongly held convictions, a tendency towards judgmental attitudes regarding MHC, and the inherent complexity of OD has resulted in some FACT professionals labeling it as anti-psychiatry. In this context, some FACT professionals reported feeling hesitant to voice any criticisms of the approach, as they experienced that and feared it could be perceived as a personal attack. Conversely, OD professionals and OD trainees stated that they often found themselves trying to convince their colleagues of the value of the approach, as they sensed that it was not fully understood. This tone of conviction and belief in OD contributed to the skepticism of some FACT professionals, who viewed them as “believers” and referred to the “hallelujah effect” of OD as though it were a panacea. These dynamics between OD professionals, OD trainees and FACT professionals created a sense of insecurity and unease for both sides during the pilot.

“There was a lot of resistance, especially against the idea of going there for a week, doing a lot of meditation, that it is important, and essentially it was just said that you are being brainwashed and joining a cult. And that's where this team encountered a lot of challenges” (OD trainee).

“[name of colleague] was there almost like a guru, a completely different person as far as I knew before. Hallelujah, how wonderful OD was and how we have all been doing it wrong so far. And that really shocked me because if you didn't attend those training weeks, you missed all of that, of course. So that's a sudden change, just like with my teammate, who came back from the training week with hallelujah, while we missed that … It is immediately taken personally, while that, I mean the criticism I have about OD, is not personal at all. I'm talking about the method. Yeah, you're actually not supposed to say that” (FACT professional).

In addition, emotional discomfort was attributable to the multitude of changes during the transitional process. Namely, a complicating factor in this pilot program mentioned by all professionals was the lack of time due to work pressure resulting from the rapid succession of changes in mental healthcare and the imposed production standard (an imposed percentage of the working time that must be billable). This made professionals (feel) overloaded. Management had chosen in this pilot program not to use a well-defined plan and to give the teams the space to experiment. However, it seemed that professionals found it difficult to experiment and push the boundaries while experiencing this work pressure. Several OD professionals and OD trainees indicated that it was too non-committal in the pilot whether network treatments are offered. To create space in this dynamic context, some OD trainees wondered whether imposing goals in the FACT teams, for example x number of network meetings per year, would help OD trainees to experiment by being able to justify to colleagues why they took the space to experiment.

“So, how do we manage to have enough conversations between [colleague’s name] and me, that’s quite a challenge… Although we have a very enthusiastic team and we try to think of creative solutions, it’s still difficult. Moreover, if we want to do more with OD, we can never fulfill that in terms of time, schedule, and availability” (OD trainee).

### The need for multi-stakeholder participation and support

As OD professionals pursued answers to policy-related questions, such as the degree to which person-centeredness should be implemented in practice, it became apparent that these inquiries do not solely pertain to OD professionals and OD trainees. Achieving significant change necessitates the participation and consent of, e.g., clients, their network, the MHC organization and (in fact) society as a whole. To illustrate their quest, we provide elaboration on some examples of policy-related questions that have implications beyond the immediate influence of professionals.

One example is related to the role of clients themselves, in which the starting point is that the OD professionals follow the client’s pace. This approach puts the client in charge of determining whether and when the next session is necessary. The OD professionals noticed that some clients did not take the initiative to contact them, which raised the question of whether it is appropriate to reach out to clients within the OD approach, given the team’s desire to maintain contact. Eventually, they arrived at a compromise and began to view contact as a shared responsibility. As part of this approach, the team periodically reviewed their careload and reached out to clients who had not been in contact for an extended period of time. They also worked collaboratively with clients who expressed a desire for contact but had difficulty initiating it, utilizing shared decision making to find an appropriate solution.

“So, you no longer make plans for someone. You really leave the choice with the client and their network… We sometimes really take risks. In the sense that if the client really does not want something, sometimes we just do not do it. Even though everything in you calls for intervening… And what you need as a team then is… a team that can just explore together how are we going to handle this, is it going to be an exception to our principles or not. Well, that asks for a lot of thinking and consultation” (OD professional).

In addition to clients, these type of policy related questions also concerns the client’s network. For example, not all professional network partners were equally enthusiastic beforehand to hear that nothing about the client is discussed without the client being present. The OD professionals had to discuss and decide in some situations to what extent they wanted to stick to the adage or make an exception to prevent the client from being duped, for example in the case of an assessment to qualify for a certain compensation. The next question was how you make sure it remains an exception. These kinds of situations mainly occurred at the beginning of the pilot.

“People had to get used to the fact that we were not simply providing information or having discussions without the client being present” (OD professional).

Another example is the difficulty and challenge all professionals experienced translating the concepts of person-centeredness care into practice in case of clients facing a (potential) crisis. In order to be able to continuously follow the client’s process during a crisis and to promote the dialogue, it is necessary that all those present in the network session tolerate the uncertainty that people experience strongly during a crisis. This requires letting go of the tendency to immediately reach for solutions. This is often difficult for the attendees, because clients and their (professional) network members often have a strong belief that there is a problem that can be solved by experts, who are expected to come up with a solution during the session. This conviction that mental problems need to and can be solved by MHC professionals seems to be deeply rooted in the Dutch society. This situation is often further complicated by the interests of participating (professional) network members: e.g. trying to avoid an admission due to a shortage of available hospital beds or family members who want to be relieved of the burden in the event of a crisis.

“Especially in difficult and challenging situations, being able to truly listen, be fully present, and consciously maintain awareness of one’s own feelings and thoughts, including bodily sensations, as well as being able to tolerate pain and sadness in oneself and others without immediately reacting, and also being open to different perspectives, voices, or opinions” (OD professional).

“And I think those interests are often just very complicated. And it often becomes complicated, in my opinion, when you have people from, for example, [name of professional network partner], who are simply sent by the municipality to minimize spending. I find that those are the moments when OD becomes most challenging” (OD professional).

“What we see in society is an increasingly convulsive attitude towards deviant behavior, or behavior that you do not understand. We seem increasingly shy to deal with or respond to this in a good way. And there’s a certain growing, I think, intolerance to behaviors that we do not understand. So that has to be solved” (OD professionals).

In addition, OD professionals underlined the importance of the board of directors of their organization to be explicit about their aspirations with OD in the organization, because the manager’s circle of influence is only within one of the organization’s departments. For example, once OD’s clients need admission due to crisis, the OD professionals need to call in crisis care which is organized within another department. They collaborate, but the client is transferred to professionals of the crisis care department who are not working in line with the OD approach. Only the board of directors has the mandate to decide to change this and ensure continuity of care in line with the OD approach.

In line with this, OD professionals and OD trainees added that the discussions on the policy level and the change needed to apply person-centered care goes beyond the mental health care and evoke broader societal questions.

“Tolerance for uncertainty, not knowing, not diagnosing, but allowing meaning to arise. Yes, well, I think that’s the most beautiful thing about the whole OD. It’s about the moment. And that’s the pitfall. There are people who sit on the couch with a family and think it’s all fine. And where will they end in 5-years? And can you tolerate that as a society? Is it okay for people to sit on the couch for 30-years?… As a society, can we tolerate that? Why do we all have to do what someone has come up with?” (OD trainee).

“Now that I have started the training? To give clients and their system the voice back that they have more or less lost due to our entire healthcare system. To let go of the thinking centered around medication and the whole medical approach. Yes. And I believe that presents a huge challenge. Yes. … For all of us. For healthcare. And for how we have organized it together. Even at the team level. On a personal level… a team would have to collaborate differently if we were to truly embrace more of the OD approach. And I think the entire organization, the financing, would also need to be organized differently in order to truly implement it” (OD trainee).

“Well, you see, I still think that approach assumes there is a very clear and distinct problem and that you will solve it with a very clear solution or treatment… And as long as we continue to think that way in the Netherlands, I expect that OD will face significant challenges” (OD professional).

So, during the pilot, OD professional and OD trainees came to the realization that the necessary changes among the professionals and team could only be fully achieved if others also move along with this change. Given the size and intricacy of the change process, all professionals expressed the need for an implementation plan by the organization. They stressed the importance of managers to be clear about the future ambition of OD (e.g., will it remain an intervention in the regular teams or will they also become OD teams). Moreover, OD trainees wanted the managers to give clearer instructions about what to do (e.g., setting a number of expected network meetings per year) to legitimize to give OD priority and to do OD within the FACT teams. In addition, the OD trainees experienced that having two OD trainees in a team is not enough to do full justice to the OD approach. Some OD trainees felt mangled by the organization after coming back from training. They came back enthusiastic but struggled to make time to put OD into practice or clashed with others’ skepticism about OD. All OD trainees believe that if no new colleagues are trained, it remains an intervention that is added to the treatment trajectory with the risk that it will fade out at some point in time. They added that in order to truly embed OD, continuous awareness of OD is needed. Moreover, in directing the change process, especially the role of psychiatrist, psychologist, manager and medical director were considered key. Additionally, it was deemed crucial to encourage polyphony among the group of professionals, encompassing diverse perspectives. This would include a well-balanced mix of professionals who are willing to experiment and those who possess a contemplative approach and are adept at asking reflective questions.

“You need to have a direction. You need to create an implementation plan… What do I need to implement? That just makes it very frustrating. Because if I come back from England all excited, yes, great. And then what? Make it clear where you need to go. And then, with the people who have been trained, let them be the ones who bring others along to implement it. But make sure you communicate what you want to implement. And how” (OD trainee).

## Discussion

The primary objective of this study is to gain insights into the challenges experienced by professionals during their transition towards a person-centered MHC approach, called OD. Our focus is on the social and inter-individual dimension of the change process that underpins a social innovation such as the introduction of person-centered care. Specifically, we reflect on the challenges that MHC professionals encountered in adopting the person-centered care philosophy. Through this social lens, we discovered in this study four challenges that MHC professionals came across when pursuing this change: (1) understanding and knowledge transfer, (2) (inter)personal process, (3) emotional discomfort, and (4) the need for multi-stakeholder participation and support.

First, we start with the challenge that strongly emerged in the findings, which involves the understanding and transfer of knowledge. The field of knowledge management provides valuable insights into the underlying complexity of this issue. Knowledge encompasses multiple dimensions and is typically classified into explicit-and implicit knowledge. While explicit knowledge is precise, observable, and tangible, it represents only a fraction of the overall nature of knowledge and expertise. Most of the knowledge capital lies in the concealed experiences, know-how, and skills of individuals, known as implicit or tacit knowledge (67). This study suggests that the same principle may apply when it comes to comprehending person-centered care concepts like OD. In fact, the “onion model” offers a valuable framework for delving into the intricate layers involved in understanding and transferring knowledge. This model offers a comprehensive perspective on tacit knowledge, viewing it as a hierarchical and dynamic phenomenon. It proposes distinct layers of tacit knowledge, each residing at various levels of consciousness: hidden practical knowledge, reflective tacit knowledge and demonstrated tacit knowledge, representing the deepest level of tacit understanding (67).

One aspect of the challenge of understanding and knowledge transfer seems to be related to the hidden practical knowledge in the first layer, which represents concrete knowledge and pertains to how professionals carry out specific actions in a person-centered practice (67). From a linguistic perspective, the complexity arises because certain words can have different meanings. Contemporary psychiatry seems to be focused on patient-centered care, which shares similarities with person-centered care in terminology (19). However, as shown by the different citations in our results, it fails to recognize that person-centered care is fundamentally different. This may explain why professionals often believe they are already working in this new way ((11, 68–71)). In this study, for example, they indicated that they already listen carefully to clients and tailor care to clients’ needs while maintaining a humble attitude. Despite both approaches emphasize the individual’s needs, preferences, and values, person-centered care takes a more holistic approach and has a different objective. It aims to help clients lead a meaningful life according to their own wishes, rather than focusing on diagnosis, treatment decisions, and functional outcomes. Person-centered care expands on the principles of patient-centered care by considering the entirety of a person’s life (19, 68). Another instance of a qualitative difference between patient-and person-centered care can be observed in the theme of communication. Articles focused on patient-centeredness commonly depict communication as the efficient and precise exchange of information. In contrast, articles emphasizing person-centeredness place greater emphasis on elucidating what truly matters to the individual, highlighting dialogue and narrative as key elements (19). To achieve this, MHC professionals need to take an additional step by viewing the client as a person with agency, influenced by social, psychological, and environmental factors. They should empathize more deeply by letting go of preconceptions, relinquishing control, and adopting a guiding role instead of an expert one (2, 19, 72–74).

The challenge of understanding and transferring knowledge extends to the second and third layers: reflective tacit knowledge and demonstrated tacit knowledge, respectively. The second layer involves greater abstraction, such as professionals’ rules of thumb for decision-making, with less conscious accessibility than the first layer. The third layer, furthest from our conscious awareness, encompasses inexpressible knowledge (67). In this study, we observed these layers’ manifestation in person-centered care, posing challenges in understanding, articulation, and transfer, often associated with “learning by doing.” This aligns with earlier literature [e.g., (67, 75, 76)]. For example, our study reveals an ongoing complexity of applying person-centered care, raising the question of the appropriate level of person-centeredness. Relying solely on a one-sided approach risks undermining professionals’ expertise (21). Achieving effective person-centered care necessitates the engagement of both parties, leading to what is known as “inter-person centered care”. This involves taking into account factors like clients’ options and the practical feasibility for professionals (6). Achieving effective person-centered care requires both parties’ involvement, leading to what is known as “inter-person centered care”. This involves taking into account, e.g., clients’ possibilities and professionals’ practical feasibility (6). Ambiguity surrounding the extent to which person-centered care should be client-centered is evident in the *literature* (9, 77, 78). Finding the right balance and embracing shared responsibility in implementing person-centered care lacks a predetermined answer. It necessitates a customized and intuitive approach, taking into account the unique circumstances of individuals and ethical dilemmas that may arise, such as managing conflicts between “risk management” and “self-determination” during crises (22). This aligns with literature highlighting the pivotal role of tacit knowledge, acquired through accumulated experiences and intuitive actions, in deepening the understanding of the concept of person-centered care beyond mere theoretical comprehension (67, 79).

Thus, OD professionals encountered challenges in comprehending the concept and encounter similar difficulties in effectively conveying it to OD-untrained professionals. The latter may not be surprising, since untrained professionals face the same obstacle, namely the difficulty to understand the concept. As a result of this complex nature of understanding and transferring knowledge about person-centered care, there was an underestimation of the profound and far-reaching impact that implementing OD had on professionals themselves and the organization of care. The extent of this impact gradually became evident, leading us to the second challenge of (inter)personal process.

Shifting our focus to the second challenge, which is the (inter)personal process, it becomes apparent that embracing the person-centered approach entails a high-impact individual journey for MHC professionals. This finding is in line with previous studies, acknowledging that it encompasses a profound transformation of each therapist’s self and practice, involving substantial adaptations in terms of professional expertise, beliefs about mental health, values, and conduct (38, 80). Each professional and team underwent a unique process of implementing person-centered care, albeit in separate groups. Interestingly, groups with similar training years formed communities, while there was a lack of interaction between these communities. This phenomenon could be (partially) attributed to cognitive and social boundaries (52) that arise from the individualized nature of the process, thus limiting shared experiences. Professionals working in multidisciplinary teams tend to seek coherence in knowledge within their own team (52). As a result, professionals often adapt to one another’s knowledge, leading to the development of group knowledge that is predominantly tacit in nature and challenging to effectively communicate or share with others. Consistent with existing literature, this study suggests that the necessary tacit knowledge is acquired through experience and transmitted through interpersonal contact within these communities. This knowledge is informal and process-oriented (67, 81, 82) and only develops over time (75). It exemplifies the saying “you cannot make the grass grow faster by pulling on it” or, as football coach Cruijff aptly said, “You only see it once you get it.”

In terms of this (inter)personal process, this study does not provide a definitive approach to navigating the transformative journey. On one hand, the findings suggest that rigorous training is crucial for undertaking such a profound personal process, consistent with previous studies (11, 83, 84). On the other hand, the study raises concerns about the potential risks associated with training methods such as family constellations, group pressure, and the intensity of the residential training. Terms like “sectarian” and “brainwashing” were used as cautionary measures against participants losing their critical thinking abilities. This concern pertains to the risk of groupthink, a theory proposed by Irving Janes, which entails the conformity of individuals to group values and ethics (85), where the challenge lies in maintaining critical thinking amidst a transformative personal journey. The question whether this concern is valid remains unanswered. The fact that OD stands for polyphony and thus embraces diversity of voices (73) may reduce the likelihood of groupthink. Additionally, the strong dedication of professionals and the inward focus that some perceive as “sectarian” may stem from an entirely different cause. It could also be attributed to the significant impact of the change in an already turbulent period within the MHC, characterized by high workloads, causing professionals to be preoccupied with their own processes and consequently less attentive to connecting with others. Regardless of the answer to this question, results of this study showed that the internalization of the person-centered care concept necessitates continuous self-work, self-care, and critical thinking.

Next, we shed light on the third complexity of emotional discomfort. In addition, and in accordance with previous studies (8, 19), we observed in this study that the transformative shift to person-centered care provoked fear and resistance among some non-OD-trained professionals. The term anti-psychiatry emerged in this study, possibly because OD indeed criticizes the current system. However, it would be an overgeneralization to categorize OD as anti-psychiatry, as OD does not oppose psychiatry but rather promotes its transformation in accordance with critical psychiatry principles (8). For example, person-centered care challenges the value-justice aspect of MHC (10), emphasizing the importance of treating clients with respect, allowing them to make their own decisions, and ensuring their voices are heard equally. It is suggested that the justification for patient-centered care in current mental healthcare is most commonly to improve treatment outcomes (10). In contrast, person-centered care emphasizes the underlying value that person-centeredness should be perceived as a fundamental starting point and a human right for every individual, irrespective of the results achieved (7). In this confrontation with the current MHC system, a similar dynamic seemed to emerge, as seen in social movements (86). Both trained and untrained professionals tend to feel personally affected quickly, possibly because these are fundamental values that are at stake. This emotional discomfort may be related to idea that our views on psychiatry serve a similar purpose to religion in people’s lives, offering guidance in understanding and managing mental illnesses. These beliefs can be held with a high level of certainty, and if challenged, may lead to anxiety. Similar to the stress associated with giving up one’s religious beliefs, abandoning the current mental illness model could create significant concern about the professional viability of MHC professionals (8).

Taking also the fourth complexity into consideration, the need for multi-stakeholder participation and support, findings are consistent with previous research findings (9, 22, 68). This study revealed that the necessary change goes beyond the team and requires change from the client, organization, mental health care system, and society as well. If current mental health care truly wants to deliver person-centered care, then addressing this level of complexity that exists within this change process is essential (9, 21, 22) and not only person-centeredness within psychiatry needs to be taken into account, but also the barriers that come with attaining person-centeredness throughout society (22).

Interestingly, our results indicate a predominant focus among professionals on the necessity of an implementation plan within the organization, with appropriate involvement of key individuals. While we recognize the importance of having an implementation plan and involving key individuals in change processes, upon careful consideration of the four interdependent challenges, it can be deduced that embedding a person-centered approach such as OD appears to constitute a so-called third-order change (87). Thus, the transformation requires a systemic shift denoted as a novel situation X, rather than “just” advancing through incremental improvement within the organization (87, 88). This notion that a third-order change seems to be underway, leading to an as-yet-unfamiliar situation X, could provide a partial explanation for the difficulties encountered in understanding and conveying the comprehensive concept of person-centered care. Furthermore, the art of change may not so much lie in directly managing the transition of the MHC system itself through an implementation plan, but above all in the ability to orchestrate the interactions among the involved stakeholders and address the associated challenges (87, 88). First, more time and attention may need to be given to creating a shared understanding (51) of person-centered care.

### Limitations and strengths

One of the limitations of our qualitative study has to do with the limited sample size of fourteen MHC professionals. This limitation resulted from a lack of resources and the scarcity of available participants due to their high workload. Despite this limitation, these fourteen participants can be considered “key informants, “providing a distinctive firsthand perspective on the subject of the study (38). Although we considered OD trainees as having both a FACT and OD perspective, it is possible that the FACT perspective is somewhat underrepresented in our study. Nevertheless, during the process of data analysis, recurring themes emerged in all interviews, which we took as a sign that data saturation was achieved after all. Another limitation may be our exclusive reliance on qualitative data. However, this is also a strength, as qualitative analyses offer the opportunity to delve into complex phenomena, gain important insights and capture the views of participants. We conducted in-depth analyses from different perspectives to uncover patterns and increase understanding of professionals’ challenges and the inherent complexity underlying this high-impact change process. Furthermore, we recognize that, despite the involvement of multiple researchers in the analytical process, the categorization of sub-themes into themes can still contain a degree of subjectivity. Thematic analysis allows for various interpretations and ways of connecting themes (89). However, this challenge is inherent in analyzing themes at higher levels of abstraction, as observed in this study. Another critical aspect to consider is that we examined a person-centered approach without incorporating the perspective of clients themselves. This is unfortunately not uncommon, as the majority of papers on person-centered care lack the client’s viewpoint. This contradiction raises the question of who is truly at the center. Excluding the client’s perspective explicitly may result in the viewpoints of researchers and MHC professionals filling the void (10). On the other hand, in order to become person-centered, MHC professionals need to change themselves. This aligns with the saying, “be the change you wish to see in the world” (often attributed to Mahatma Gandhi) or as succinctly stated in the postgraduate OD training program in the UK, “It’s not about them, it is about us, “referring to the notion that MHC professionals must take the first step in making MHC more receptive to the person-centered philosophy. Building upon this train of thought, we hope that this study contributes to a better understanding of the challenges that MHC professionals may encounter during the transition towards person-centered care and the underlying complexities of these interrelated challenges.

### Conclusion and future research

This study delved into the challenges that arose during the introduction of OD as a person-centered approach in a MHC ambulatory setting. We found that introducing a person-centered approach, such as OD, in current MHC goes beyond implementing specific practices or procedures. It requires a broader discourse on the underlying values and human rights that guide such an approach. It is possible that in current efforts towards implementation of the person-centered OD approach, the significance of making MHC more receptive to these underlying values may be overlooked or underestimated. By initiating an open dialogue among stakeholders, while embracing the existing polyphony, and fostering a comprehensive and shared understanding of the underlying values (the why) and their practical multiple-layered implications (the how), it becomes possible to take an important initial step towards addressing the complexities involved in implementing a person-centered approach, such as OD. We hope this study makes a valuable contribution to this broader essential discussion.

However, further research is needed to support this transformation. Future research could contribute to this development by gaining insight into how MHC professionals could deal with these complexities. Additionally, in the realm of knowledge transfer, there is value in gaining a deeper comprehension of how to effectively convey this tacit knowledge within the context of MHC. Despite certain steps forward, much remains to be done, as complex challenges require comprehensive solutions. This means for example that to effectively tackle the multifaceted issues surrounding the implementation of person-centered mental care, it becomes imperative to consider not just person-centeredness within the field of psychiatry, but also the obstacles inherent in fostering person-centeredness throughout society; in our organizations, families and communities (22). The imperative is to promote a more “people-centered” approach (90). In upcoming research, there should be a concentrated effort to delve into questions such as how clients and their (in) formal networks perceive person-centered care. This will provide insights into their struggles and needs. It is crucial to remember that these individuals lie at the core of care, but it is not about “us” or “them,” but about people with shared responsibility, everyone’s voice matters.

## Data availability statement

The datasets presented in this article are not readily available because the raw data supporting the conclusions of this article cannot be made completely anonymous. Requests to access the datasets should be directed to KL-A, c.a.g.lorenz@tilburguniversity.edu.

## Ethics statement

The studies involving humans were approved by Dutch Ethical Review Board of Tilburg School of Social and Behavioral Sciences, Tilburg University (REF EC-2019.EX113). The studies were conducted in accordance with the local legislation and institutional requirements. The participants provided their written informed consent to participate in this study.

## Author contributions

KL-A, JB, and IB: conceptualization, methodology, and writing—review and editing. KL-A: thematic analysis and writing—original draft preparation. KL-A and JB: analysis, interpretation of results, and project administration. JB and IB: supervision. All authors contributed to the article and approved the submitted version.

## Conflict of interest

The authors declare that the research was conducted in the absence of any commercial or financial relationships that could be construed as a potential conflict of interest.

## Publisher’s note

All claims expressed in this article are solely those of the authors and do not necessarily represent those of their affiliated organizations, or those of the publisher, the editors and the reviewers. Any product that may be evaluated in this article, or claim that may be made by its manufacturer, is not guaranteed or endorsed by the publisher.

## Supplementary material

The Supplementary material for this article can be found online at: https://www.frontiersin.org/articles/10.3389/fpsyt.2023.1250856/full#supplementary-material

## Abbreviations

OD: Open Dialogue
MHC: Mental health care
MH: Mental health
FACT: Flexible Assertive Community Treatment
NPT: Normalization Process Theory
SMI: severe mental illness
