# Supplementary material for: Unraveling complexity in changing mental health care towards person-centered care
Source: Front Psychiatry. 2023 Sep 14;14:1250856. doi: 10.3389/fpsyt.2023.1250856 (PMC10536252; doi:10.3389/fpsyt.2023.1250856)
Supplement: Supplementary file 1 [file Data_Sheet_1.pdf]

Appendix I: How person-centered care takes shape within OD

In this appendix, we depict how OD professionals described how OD embodies person-centered care. In the OD approach, the client's network is brought together in network treatment sessions, from the start of the treatment process. These sessions serve as the basis of the entire treatment process. OD professionals added that their attitudes and interactions with clients and their network have undergone significant changes to align with the person-centered OD approach. These changes mentioned are evident in various ways, such as shared-decision that prioritizes the client's perspective, a focus on the client’s network rather than just the individual client, prioritizing dialogue and relationship-building over problem-solving, and promoting self-determination and equal partnership.

Related to shared-decision making, OD professionals described the adage 'nothing about me, without me' as the starting point of the new practice. They saw this as a fundamental shift in approach, as it means that nothing is discussed about or decided for the client without the client's presence and involvement. As a result, OD professionals no longer held experts meetings to discuss clients without client being present. Another result was that, through shared-decision making with the voice of the client as the final decision maker, it was decided who was invited to each session, and relevant professionals are invited as well, if specific expertise is needed.

With regard to the focus on the entire network, OD professionals explained that two or more OD professionals are present during the network treatment sessions to listen to the stories of the client and network members, to promote open communication and polyphony, and to facilitate dialogue between client and network members. If a crucial person in the client's network cannot attend, the client is often asked during the session to share what they think that person would have said. All contributions are considered equally important and the focus is on the entire network rather than just the individual seeking help.

In relation to their primary concern, OD professionals explained that they - in contrast to traditional MHC - do not initially focus on diagnosing the client to determine the best course of treatment or to solve the problem. They said that the sessions typically last an average of 1.5 hours, which is double the duration of regular treatment sessions, allowing sufficient time to thoroughly explore the situation without seeking a quick solution. Their primary concern is establishing contact and fostering dialogue, which is an ongoing process that can provide new insights and perspectives each time.

With respect to facilitating self-determination and equal partnership, the OD professionals emphasized that OD professionals do not have personal motives during the network treatment sessions and guide the network without directing it in any specific direction. In line with this, OD professionals do not review treatment records or set an agenda before the start of the session, meaning that the starting point of the session is not determined by the previous session's topic, professional concerns, or pre-defined treatment goals. They explained that sessions start with a simple question of how each attendee would like to use the time. The OD professionals follow the lead of the attendees by responding to their utterances and asking open-ended questions, avoiding the insertion of interpretations or expectations. They added that during these sessions, there are moments known as reflection moments, where OD professionals turn to each other and share their thoughts and physical experiences such as emotions or physical reactions, to increase the group's awareness of what is happening in the session. The other attendees listen without responding at this time. After the reflection moments, the client and network members are given the opportunity to respond if they

choose to do so. In this way, they share their insights and ideas, but do not provide direct advice. In other words, they bring their expertise to the table, but do not present themselves as the ultimate experts. OD professionals explained that all attendees of the sessions share responsibility for the process this way.

In addition, in the learning process towards this person-centered approach, all OD professionals have found that practicing mindfulness helps to make the needed transformation as a professional. This mindfulness practice, which they do for example through daily (guided) meditation, helps them overcome the urge to analyze and steer the conversation, and to instead stay present and listen. So they apply mindfulness practice as a tool to learn to enhance their present-moment focus, to become more aware of their thoughts, feelings, and sensations in a non-judgmental way and increase their attention control. This (self-)awareness allows them to focus on the present moment and to recognize when they are getting caught up in their thoughts and helps to consciously choose to step back from these thoughts. In this manner they learn to focus their attention on the dialogue between the attendees of the treatment network session rather than their own thoughts.

Classification: Internal
