# Supplementary material for: Unraveling complexity in changing mental health care towards person-centered care
Source: Front Psychiatry. 2023 Sep 14;14:1250856. doi: 10.3389/fpsyt.2023.1250856 (PMC10536252; doi:10.3389/fpsyt.2023.1250856)
Supplement: Supplementary file 2 [file Data_Sheet_2.pdf]

Appendix II: The applied implementation strategies

Our starting point is when the department manager and several colleagues introduced the concept of OD to the department and invited all professionals from the seven FACT teams to apply for the one-year postgraduate OD training program titled 'Peer-supported Open Dialogue, Social Network, and Relationship Skills' at the Academy of Peer-supported Open Dialogue (APOD) in the UK. To be eligible for the training, at least two professionals from the same team had to register. Based on motivation letters, the manager subsequently selected seven professionals to participate in the training in the year preceding the pilot, and they completed the training.

This training program consisted of four five-day residential modules. The residential setting offers the opportunity and is intended to nurture robust connections among participants. Across these modules, the emphasis lies on underlying theories of OD (e.g., Social constructionism, dialogical and reflecting processes), experiencing OD, and mindfulness and self-work, bolstered by (therapeutic) techniques such as the fish bowl technique and family constellations. To guide trainees through the sometimes-emotional journey, small support groups are arranged, affording them time after each study day to privately convene, reflecting on their learning and difficulties. Teaching staff was also accessible upon trainees' request. Furthermore, trainees were expected to engage in self-work and to submit corresponding assignments during the periods between the modules.

During this first year of training prior to the pilot, the manager has lobbied for OD inside and outside the organization. To raise awareness of OD and create support, several presentations about OD were held for clients, their network and other professionals throughout the pilot. OD professionals also used the existing communication structures in the organization to talk about OD (e.g. the psychiatrist at the psychiatrists' staff and peer-support worker at the peer-support workers' staff).

The original idea was to introduce OD in the organization by training 10 professionals from different teams in the department every year. However, halfway through this first year of training, the trainees came to the conclusion that they need to form a OD team to successfully apply the OD approach in practice. In order to achieve this, a plan was made within 6 weeks how to transform one of the FACT teams into a OD team. For pragmatic reasons, the team was chosen where one of the OD professionals is in charge of treatment. In addition, a grant application has been submitted to train more professionals and has been awarded.

After subsidy was granted and the seven professionals completed the OD training, the start of the new OD team marked the start of the pilot at the end of 2017. The team members of the former FACT team could choose to stay and follow the OD training or to move to another team within or outside the department. The team took over the caseload of the FACT team and each client was informed about the change and what it meant for them. Clients had the choice of joining OD, moving to another team or staying on the team and maintaining one-on-one contact, just like they used to. All new clients received OD care as a matter of course. The team proceeded as described in appendix I. At the beginning of each OD treatment path or when a client invited a new network member into the treatment session, OD professionals explained the procedure. In the meantime the second half of the team followed and finished the OD training in this first year of the pilot.

At the end of this first pilot year, new professionals were recruited for the training in the same manner as the first selection round. In the second pilot year, four trainees of two FACT teams attended the OD training while simultaneously introducing OD to their FACT teams. These two teams were not fully transformed into OD teams. The OD trainees could not fully adhere to the OD method, but they shared the same vision, therapeutic stance and -technique within the network treatment sessions as the OD team. In these FACT teams, OD network meetings are offered as an add on intervention to clients' regular treatment trajectory. Only plannable, i.e. no acute, network treatment sessions were feasible. OD trainees offered the opportunity for network treatment sessions to clients in their own caseload. Besides that, all FACT colleagues could request a OD network treatment session, at which they themselves were present as a network member of the client. One team has opted to make weekly hours available for treatment planning at fixed times. The other team scheduled network treatment sessions upon request. At the beginning of each series of OD network treatment sessions, OD trainees explained the working procedure of the network sessions as described in appendix I.

During the pilot, all OD professionals and OD trainees continuously trained the dialogical and related skills: the OD professionals through weekly peer-to-peer consultation and the OD trainees kept in touch in a group app, met each other between training weeks, practiced with network meetings and participated in each other's network meetings to learn from each other. In addition, OD professionals and OD trainees could participate 4 times a year in half-day refresher meetings, organized by the Dutch national OD network, in which various OD-related topics were discussed and an opportunity was given to ask questions and engage in discussion with each other. No use was made of supervision from the postgraduate training program due to limited (financial) resources. However, some OD professionals attended the mentor training program within the same postgraduate training program. Subsequently, they were able to mentor colleagues including providing supervision.

This is a provisional file, not the final typeset article
Classification: Internal
