# Supplementary material for: Unraveling complexity in changing mental health care towards person-centered care
Source: Front Psychiatry. 2023 Sep 14;14:1250856. doi: 10.3389/fpsyt.2023.1250856 (PMC10536252; doi:10.3389/fpsyt.2023.1250856)
Supplement: Supplementary file 3 [file Data_Sheet_3.pdf]

Appendix III Matrix based on Normalization Process Theory (NPT)

Normalization Process Theory (NPT) coding framework used to qualitatively analyze data pertaining to the challenges faced during the implementation of Open Dialogue (OD) in practice. This framework is based on the approach utilized by Mair and colleagues (2012) (46).

**1. COHERENCE** (Sense-making work)	**2. COGNITIVE PARTICATION** (Relationship work)	**3. COLLECTIVE ACTION** (Enacting work)	**4. REFLEXIVE MONITORING** (Appraisal work)
1.1 Differentiation<br>Is there a clear understanding of the distinctions between OD and current practices?	*2.1 Initiation*<br>Are key individuals willing to drive the OD implementation?	*3.1 Interactional workability*<br>Are individuals able to enact OD and operationalize its components in practice?	*4.1 Systematization*<br>How are effectiveness and usefulness of OD identified and measured through formal and/or informal evaluation methods?
1.2 Communal specification<br>Is there a shared understanding regarding the objectives and expected advantages of OD?	*2.2 Enrollment*<br>Do individuals hold the belief that OD should be part of their work and that they can actively contribute to the implementation of OD?	*3.2 Relational integration*<br>Do individuals have confidence in OD and each other?	*4.2 Communal appraisal*<br>How do individuals collectively assess the value of OD?
1.3 Individual specification<br>Is there a clear understanding of individual's designated tasks and responsibilities in OD implementation?	*2.3 Legitimation*<br>Do individuals belief it is right for them to be involved?	*3.3. Skill set workability*<br>Do individuals believe that OD is entrusted to involved professionals with the right mix of skills and training to carry it out?	*4.3 Individual appraisal*<br>How do individuals appraise the effect of OD on them and their work environment?
1.4 Internalization<br>Is there an understand of the value, benefits and importance of OD?	*2.4 Activiation*<br>Can individuals maintain their support for OD and work together to identify the required actions and procedures to sustain the progress of OD implementation?	*3.4. Contextual Integration*<br>Is there sufficient support from the organization?	*4.4 Reconfiguration*<br>Do individuals try to alter the MHC service?

Classification: Internal
